# **Procaine penicillin alters swimming behaviour and physiological parameters of*Daphnia magna***

**DOI:** 10.1007/s11356-019-05255-2

**Published:** 2019-05-04

**Authors:** Adam Bownik, Brygida Ślaska, Justyna Bochra, Katarzyna Gumieniak, Kinga Gałek

**Affiliations:** Institute of Biological Basis of Animal Production, Faculty of Biology, Animal Science and Bioeconomy, University of Life Sciences in Lublin, Akademicka 13 Str, 20-950 Lublin, Poland

**Keywords:** Procaine penicillin, *Daphnia*, Swimming behaviour, Heart rate, Thoracic limb activity, Oxygen consumption

## Abstract

Procaine penicillin (PP) is a β-lactam antibiotic widely used in human and veterinary medicine. Although PP is detected in surface water, little is known on its effects on aquatic invertebrates. Our aim was to determine the influence of PP on swimming behaviour (track density, swimming speed, turning angle, hopping frequency) and physiological activity (oxygen consumption, heart rate, thoracic limb movement) of a freshwater invertebrate *Daphnia magna* exposed to PP at concentrations of 11.79 mg/L, 117.9 mg/L and 1179 mg/L for 2 h and 24 h. The results showed no mortality; however, reduction of swimming activity manifested by the decreased track density, swimming speed and turning angle noted in *Daphnia* exposed to all the concentrations of PP. Increase of oxygen consumption was observed after 2-h exposure; however, decrease of this parameter was found after 24 h. PP also reduced heart rate and thoracic limb movement in a concentration-dependent manner. The results suggest that the antibiotic should not induce mortality; however, it may affect swimming behaviour and physiological parameters of *Daphnia magna* particularly inhabiting aquaculture facilities with intensive antibiotic treatment. On the basis of the present results, we also suggest higher sensitivity of behavioural and physiological parameters of cladocerans than the commonly used endpoints: mortality or immobilisation and their possible application as a part of early warning systems in monitoring of surface water toxicity.

## Introduction

Pharmaceuticals used in human and veterinary medicine are frequently detected in the aquatic environment or in the effluents from water treatment plants (Richardson and Brown 1985; Moulin et al. 2008). The main sources of these chemicals are industrial, hospital and municipal wastewaters. It is estimated that toxicity of hospital wastewater containing a mixture of different compounds is higher than that of the urban wastewater (Laquaz et al. 2018). Most pharmaceuticals possess high biological activity; therefore, they may induce various ecotoxicological effects. Antibiotics are one of the most commonly used drugs that may be transferred from pharmaceutical industry, municipal and hospital wastewaters and treatment plants to the aquatic environment (Rizzo et al. 2009; Basha et al. 2011; Kim et al. 2018). Since these compounds are only partially decomposed by ozonation and other methods (Arslan-Alaton and Caglayan 2006), they may be accumulated in surface waters. Antibiotics are known to induce toxicological effects in various aquatic organisms (Park and Choi 2008; Havelkova et al. 2016). For example, amoxicillin may induce slight toxic changes in hemocyte parameters of bivalves *Ruditapes philippinarum* and *Mytilus galloprovincialis* (Matozzo et al. 2016). In another study, florfenicol, one of the most commonly used antibiotic in aquaculture, was noted to cause a significant inhibition of cholinesterase activity in a bivalve *Corbicula fluminea* (Guilhermino et al. 2018) and suppression of hepatopancreatic antioxidant system in the gazami crab *Portunus trituberculatus* (Ren et al. 2017). Some antibiotics were also reported to induce toxic effects in a freshwater crustacean *Daphnia magna* such as lethality, inhibition of growth and reproduction in addition to bioaccumulation (Wollenberger et al. 2000; Martins et al. 2013; Kim et al. 2014; Kim et al. 2017; Huang et al. 2014; Dalla Bona et al. 2016; Ribeiro et al. 2018).

Procaine penicillin (PP) is a combination of a β-lactam antibiotic benzylpenicillin and procaine, a local anaesthetic used in human and veterinary medicine for treatment of certain bacterial infections mastitis, syphilis, anthrax, diphtheria and pneumococcal pneumonia (Crowe et al. 1997; Wright 1999; Taponen et al. 2003; Bazakis and Weir 2018). Although PP is one of the most commonly used antibiotics in the USA and Europe (Moulin et al. 2008), a number of side effects in the central nervous system in both humans and animals including seizures and other abnormalities were described (Paine Jr 1978; Araskiewicz and Rybakowski 1994). This drug was also demonstrated to affect the nervous system of aquatic invertebrates. For example, a convulsing effect due to the interference with membrane conductance to chloride was observed in PP-treated California sea hare *Aplysia californica* (Pellmar and Wilson 1977).

Although some authors described acute toxicity of β-lactams (Ribeiro et al. 2018), little is known on the effects of PP on behavioural and physiological effects in cladocerans. *Daphnia magna* is a common, invertebrate model used for toxicological testing (Manakul et al. 2017). However, the overwhelming majority of these studies show results based on determination of only two distinct and rather insensitive endpoints: mortality and immobilisation. It is noteworthy that daphnids possess a variety of more responsive behavioural and physiological parameters that may be used for early determination of toxicity (Campbell et al. 2004; Lovern et al. 2007; Bownik 2017). Some approaches have been made to use video techniques of determination *Daphnia* swimming activity; however, because of its complexity, support of digital analysis is required for quantification and differentiation between the parameters. The aim of our work was to evaluate the influence of PP on *Daphnia magna* swimming behaviour parameters such as track density, swimming speed, turning angle, hopping frequency with the use of digital analysis and physiological endpoints: oxygen consumption, heart rate and thoracic limb activity.

## Material and methods

### Animal culture

*Daphnia magna* were cultured in a parthenogenetic reproduction started from a single female hatched from ephippium according to Microbiotests Inc. The culture was maintained for several generations in 6 L tanks with 5 L of aerated culture medium under light: dark period of 16 h: 8 h with a constant temperature of 21 ± 3 °C. The medium was prepared according to ASTM standards (American Society of Testing and Materials 1986) with the following parameters: pH 7.9 ± 0.3, oxygen concentration 9.2 ± 0.4 mg/L, conductivity 380 μS/cm and a temperature of 21 ± 3 °C before the experiment. *Daphnia* were fed once daily with 5 ml/tank of baker’s yeast suspension (10 mg/L). Neonate organisms (≤ 24 h old) were used in the study and were not fed 24 h before and during the exposure to PP. Each experiment was performed in triplicate. The non-treated control *Daphnia* were kept in culture medium only.

### Chemicals and experimental design

Procaine penicillin G (1000 U/mg) was purchased from Polfa-Tarchomin. Due to low acute toxicity of the antibiotic according to Pfizer® Material Data Safety (LC50 for *Daphnia magna* > 1000 mg/L), in order to observe distinct effects, it was diluted in *Daphnia* culture medium to the following concentrations: 11.79 mg/L, 117.9 mg/L and 1179 mg/L. Turbidity of the medium containing the highest PP concentration was 8 ± 0.6 NTU (Nephelometric Turbidity Unit). Behavioural biomarkers (swimming track density, swimming velocity, turning angles, hopping frequency) and physiological parameters (oxygen consumption, heart rate, thoracic limb activity) were determined after 2 h and 24 h of the exposure to the antibiotic at the above-mentioned concentrations. The control daphnids were kept in clean medium only. All experiments were done in triplicate.

### Swimming speed

Swimming speed of *Daphnia magna* neonates was determined according to the method by Bownik et al. (2018). Ten daphnids were placed in the observation dish (ϕ55 mm) containing 10 mL of medium with the appropriate concentration of PP. Video clips with swimming animals for each experimental group were recorded for a minimum of 1 min by using a digital camera installed on a stand. As the depth of the solution in the observation dish was small, vertical swimming of crustaceans was negligible. A frame-by-frame method supported by Tracker® 4.11.0 software was used for the analysis of swimming speed of *Daphnia*. The swimming track left by a single crustacean (interpreted as a mass point) and mean velocity (*v*) expressed in millimetres per second was measured by clicking with the cursor on *Daphnia* image in each separate frame of the clip. Since the experimental animals were swimming only in two dimensions, analysis of trails was based on *x* and *y* coordinates. The average speed of an individual daphnid in each experimental group was calculated by the software and plotted as an amplitudogram. The amplitudograms for 10 daphnids were then superimposed and presented in one graph. The mean speed of 10 individual daphnids was treated as a result for each experimental group.

### Imaging of swimming track density

Swimming track density was determined by graphic analysis of images of trails left by 10 daphnids during 1-min video recording and further processing with the use of Tracker® 4.11.0 (Bownik et al. 2018; Bownik et al. 2019). The result of the analysis was an image showing swimming tracks marked with different colours. The image was then transformed to 1 colour depth with Toupview 3.7 software to black and white image in which all the tracks was shown as black pixels. The percentage of black pixels from each experimental group was then calculated from the histogram.

### Turning ability

Turning ability of swimming *Daphnia magna* was determined by evaluation of their change of angle with Tracker® 4.11.0 software. In brief, a 2-dimensional coordinate system was established for the observation dish seen in the video clips for each experimental group. The result of swimming track analysis was a graph showing turning angle (θ) visible in the *y* axis of the graph against time (*t*) on *x* axis. Turning angle of 10 daphnids in each experimental group was calculated by the software and the final result was plotted on the graph. Change of angle measuring the range of daphnid turning ability was calculated by subtraction of the minimal θ from maximal θ value for an individual daphnid and the data from 10 individual daphnids from each experimental group were meaned.

### Hopping frequency

Hopping frequency of swimming experimental organisms was determined by a digital analysis of video clips with a frame-by-frame analysis. The number of characteristic “jumps” was counted and expressed per minute.

### Oxygen consumption

Oxygen consumption was determined with Oxygraph plus system (Hansatech Instruments) according to previously published methods (Soucek 2006; Soucek et al. 2010) with some modifications. 10 daphnids were transfered to the oxygraph electrode chamber containing 1 ml of medium with appropriate concentration of the antibiotic and the oxygen consumption was measured for 30 min. The oxygen was detected by an electrode mounted at the bottom of the chamber and the signal from the electrode was transferred to a computer. Oxygen consumption rate was calculated by computer software, O_2_view version 2.09. The experiment was done in duplicate.

### Heart rate and thoracic limb activity

Physiological parameters heart rate and thoracic limb movement were determined by a frame-by-frame digital analysis of video clips recorded from a light microscope. Briefly, a single daphnid was transferred from the experimental dish in a 50 μL droplet of appropriate experimental solution to a microscope slide and the microscopic view of the daphnid was recorded for more than 1 min (with a speed of 30 frames per second) with a digital camera Nikon D3100 mounted on the microscope. The two physiological parameters were analysed by a frame-by-frame method with a multimedia player software. Separate heart contractions, thoracic beats were counted for 1 min.

### Statistical analysis

Statistical analyses were performed using Statistica® 13.1 software. Data normality and homogeneity of variances were calculated by the Shapiro-Wilk and Levene’s tests, respectively. The comparisons of means between the experimental groups were determined by one-way ANOVA followed by the post hoc Tukey’s *p* < 0.05. The results are shown as means ± standard deviation (SD).

## Results

### Swimming track density

**Fig. 1:**
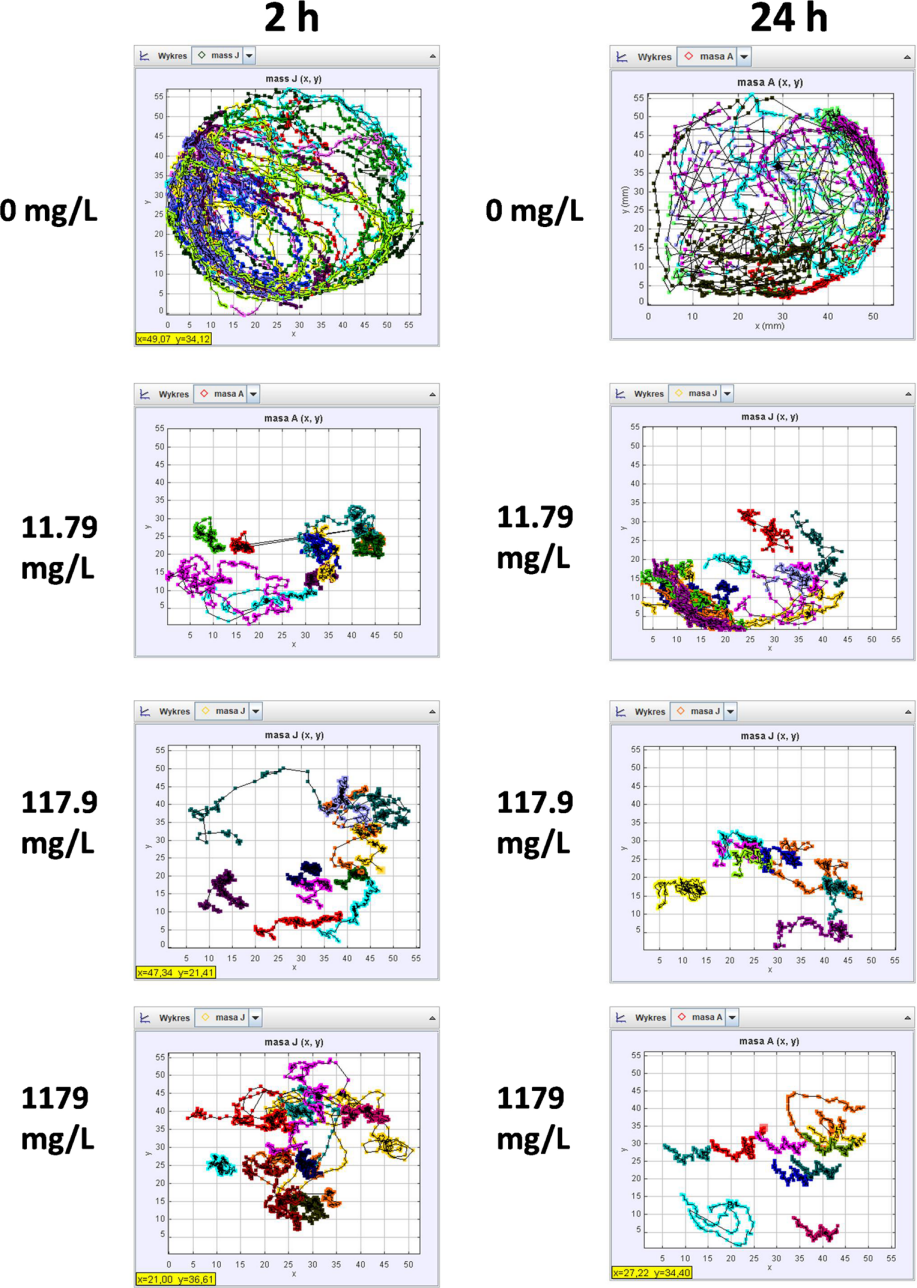
Swimming track density

**Fig. 2:**
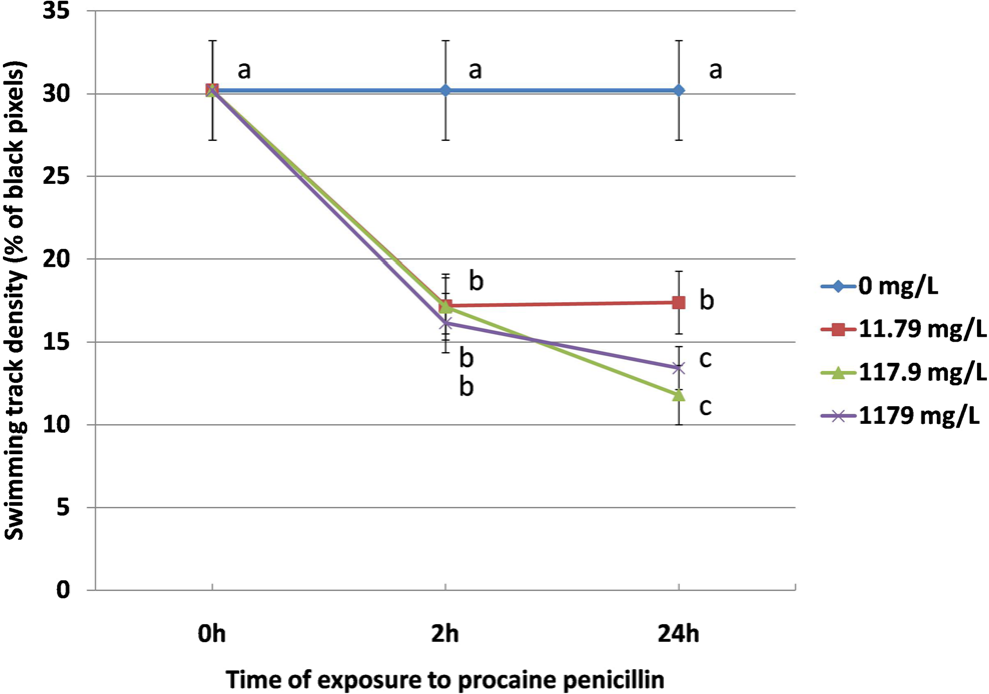
Swimming density

The experimental animals exposed to PP showed alteration of swimming track density. Images obtained with the software show that the highest density of the tracks manifested the untreated animals (Fig. 1). Daphnids exposed to the antibiotic manifest tracks of lower density. The parameter was decreased at all the concentrations after 2 h of the exposure (17.2 ± 1.7% black pixels b.p. 17.12 ± 2% b.p. and 16.15 ± 1.8% b.p. at 11.79 mg/L, 117.9 mg/L and 1179 mg/L, respectively) when compared to the untreated group (30.2 ± 3% b.p.) (Fig. 2); however, no significant differences were found between the three concentrations. Further reduction of the track density was noted after 24 h at concentrations of 117.9 mg/L (11.8 ± 1.8% b.p.) and 1179 mg/L (13.42 ± 1.3% b.p.). However, no alteration of the parameter was observed at 11.79 mg/L.

### Swimming speed

**Fig. 3:**
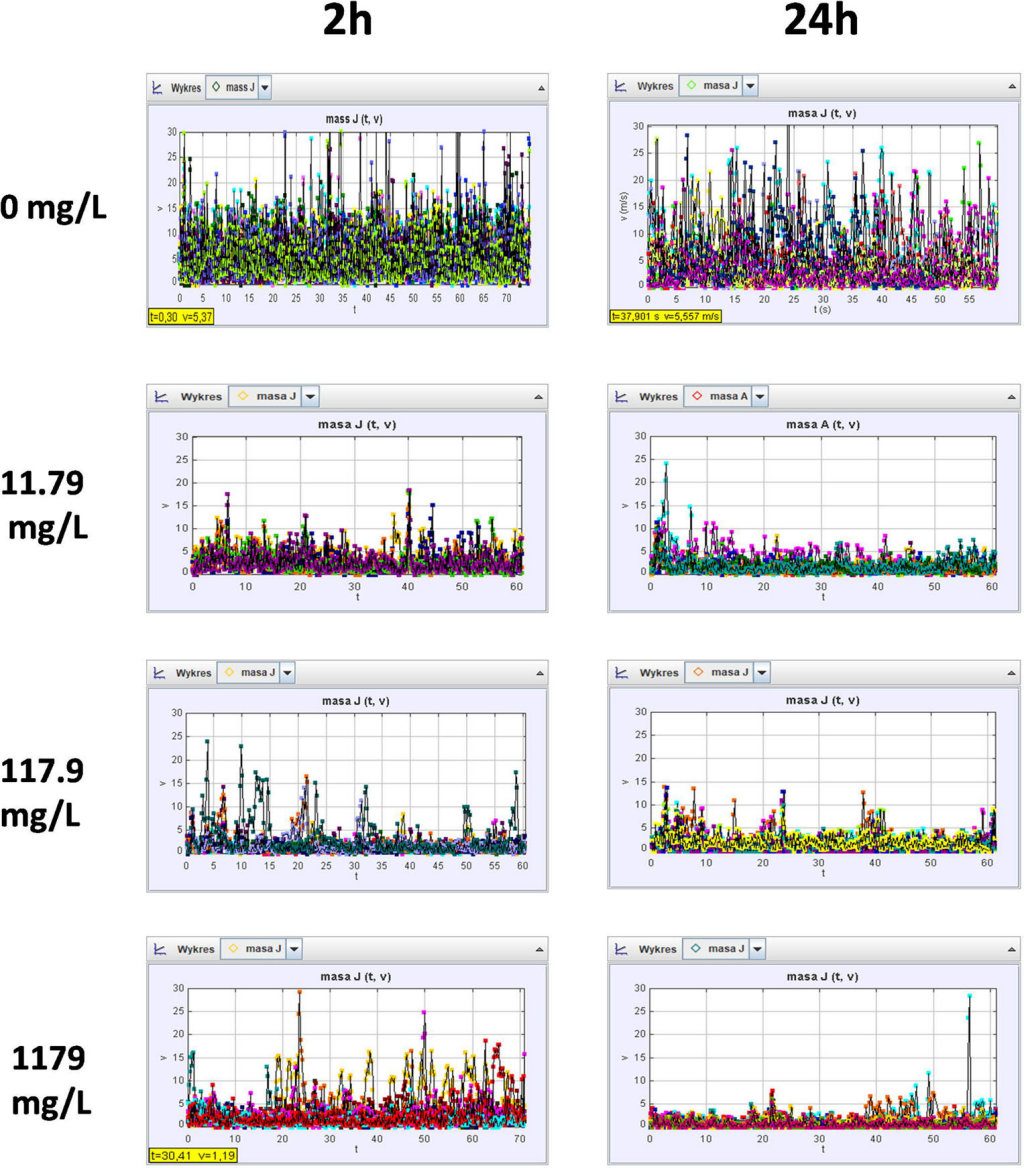
Amplitudograms

**Fig. 4:**
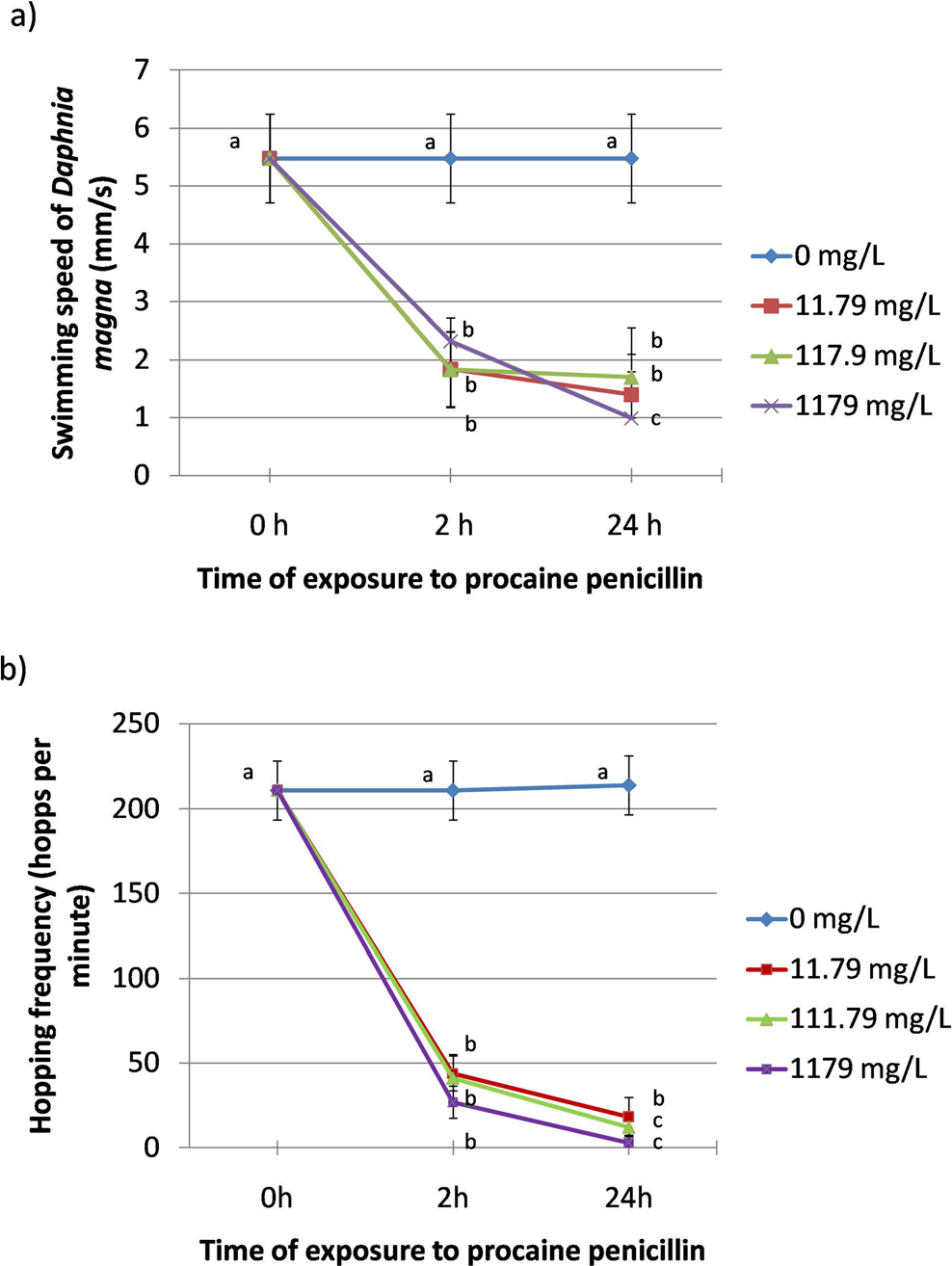
Swimming speed and hopping frequency

Amplitudograms in Fig. 3 obtained by Tracker® software show lower values of average speed for ten daphnids at each concentration of PP when compared to untreated control animals. Exposure for both 2 h and 24 h to 1179 mg/L of PP resulted in a considerable reduction of swimming speed (2.32 ± 0.4 mm/s and 0.99 ± 1.57 mm/s, respectively) in comparison to the control (5.48 ± 0.77 mm/s) (Fig. 4a). Similar results were observed in the animals exposed to lower concentrations of PP (117.9 mg/L and 11.79 mg/L) and no significant differences were noted between the experimental groups after 2 h and 24 h of the exposure.

### Hopping frequency

Daphnids exposed to 1179 mg/L of PP showed the highest suppression of hopping frequency both after 2 h (27 ± 9.5 hops per minute, h.p.m.) and 24 h (2.9 ± 0.7 h.p.m.) when compared to the control (211 ± 17.5 h.p.m.) (Fig. 4b). A considerable inhibition of this parameter was also noted after 2 h at lower concentrations of the antibiotic (44 ± 10.4 h.p.m. and 41 ± 14 h.p.m at 11.79 mg/L and 111.79 mg/L of PP, respectively) and the highest reduction at these two concentrations was found after 24 h (18 ± 0.7 h.p.m. and 12 ± 0.6 h.p.m. at 11.79 mg/L and 111.79 mg/L of PP, respectively).

### Turning ability

**Fig. 5:**
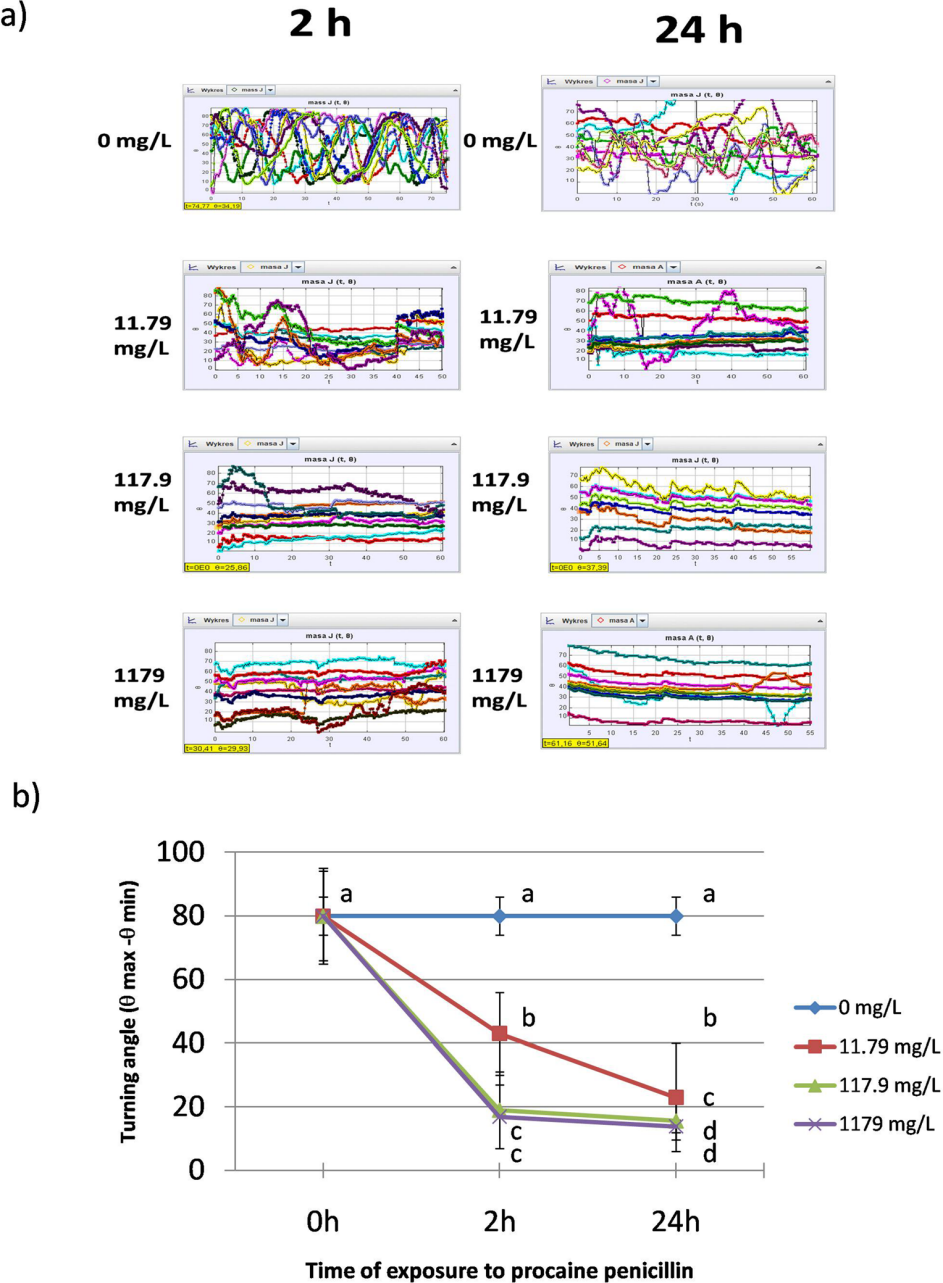
Turning angle

Images obtained by Tracker® showed that PP affected turning angle of *Daphnia magna* (Fig. 5a). Curved trails shown in the image of the control daphnids (0 mg/L) suggest their high turning angle. On the contrary, the trails left by the animals treated with the antibiotic show low turning ability. Figure 5b shows that turning angle (θ max–θ min) was decreased by PP in a concentration-dependent manner. A considerable inhibition of this endpoint was noted after 2 h at all the concentrations of PP (43 ± 13, 19 ± 12 and 16.9 ± 10 at 11.79 mg/L, 117.9 mg/L and 1179 mg/L, respectively) when compared to the untreated daphnids (80 ± 6). The parameter was more depressed after 24 h (23 ± 17, 15.6 ± 6 and 3.9 ± 2 at 11.79 mg/L, 117.9 mg/L and 1179 mg/L, respectively).

### Oxygen consumption rate

**Fig. 6:**
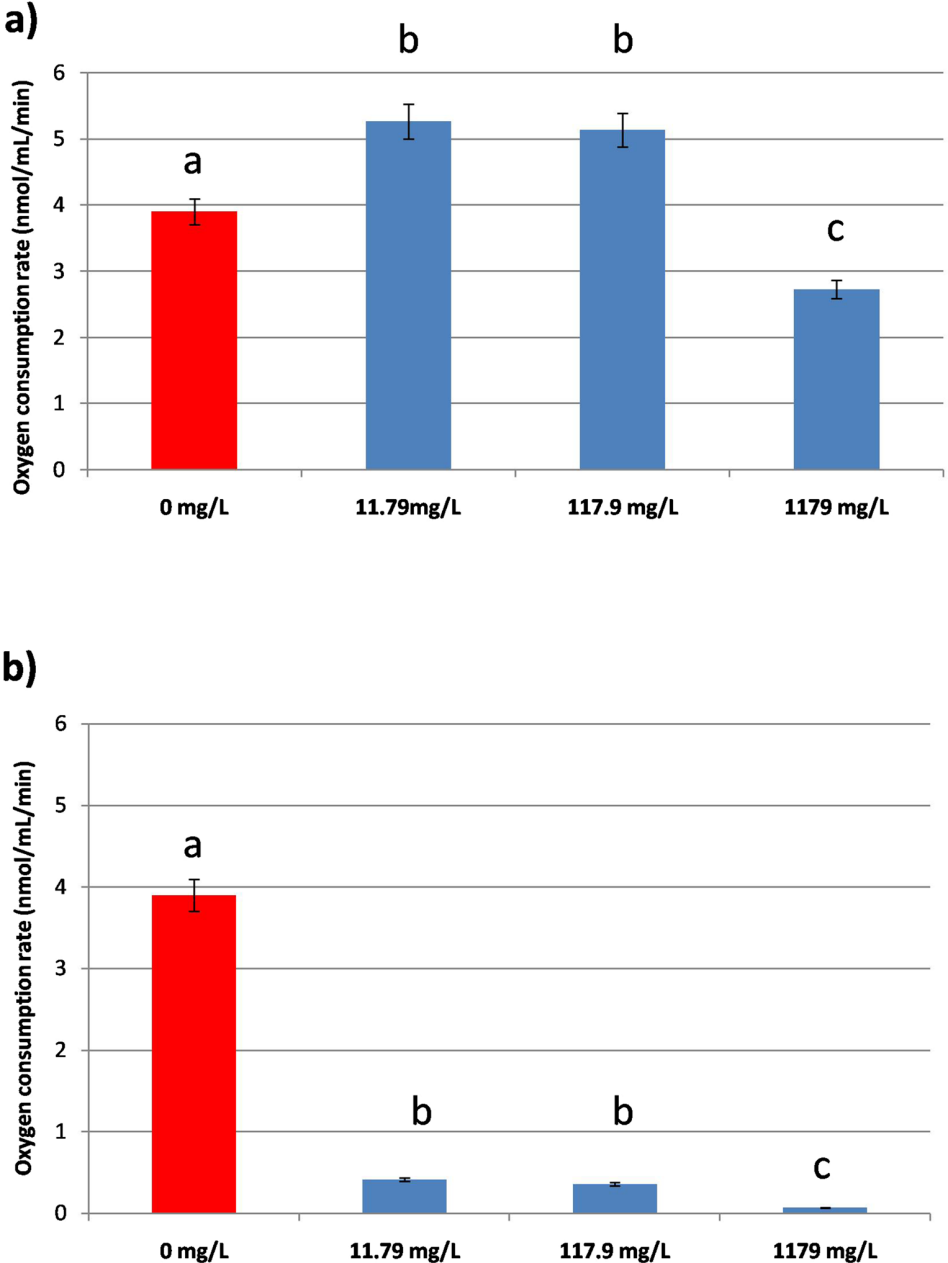
Oxygen consumption

PP induced alterations of *Daphnia magna* consumption rate. The animals exposed for 2 h to concentrations of 11.79 mg/L and 117.9 mg/L of the antibiotic showed stimulated this endpoint (5.26 ± 0.3 nmol/min/L and 5.13 ± 0.4 nmol/min/L, respectively) when compared to the control daphnids (3.9 ± 0.2 nmol/min/L) (Fig. 6a). However, treatment with the highest concentration of PP resulted in the inhibition of the parameter (2.7 ± 0.15 nmol/min/L). Twenty-four-hour exposure to the antibiotic induced inhibition of oxygen consumption rate at all the concentrations used (0.412 ± 0.024 nmol/min/L, 0.358 ± 0.015 nmol/min/L and 0.07 ± 0.001 nmol/min/L at concentrations of 11.79 mg/L, 117.9 mg/L and 1179 mg/L, respectively) when compared to the untreated daphnids (1.055 ± 0.02 nmol/min/L) (Fig. 6b).

### Heart rate

**Fig. 7:**
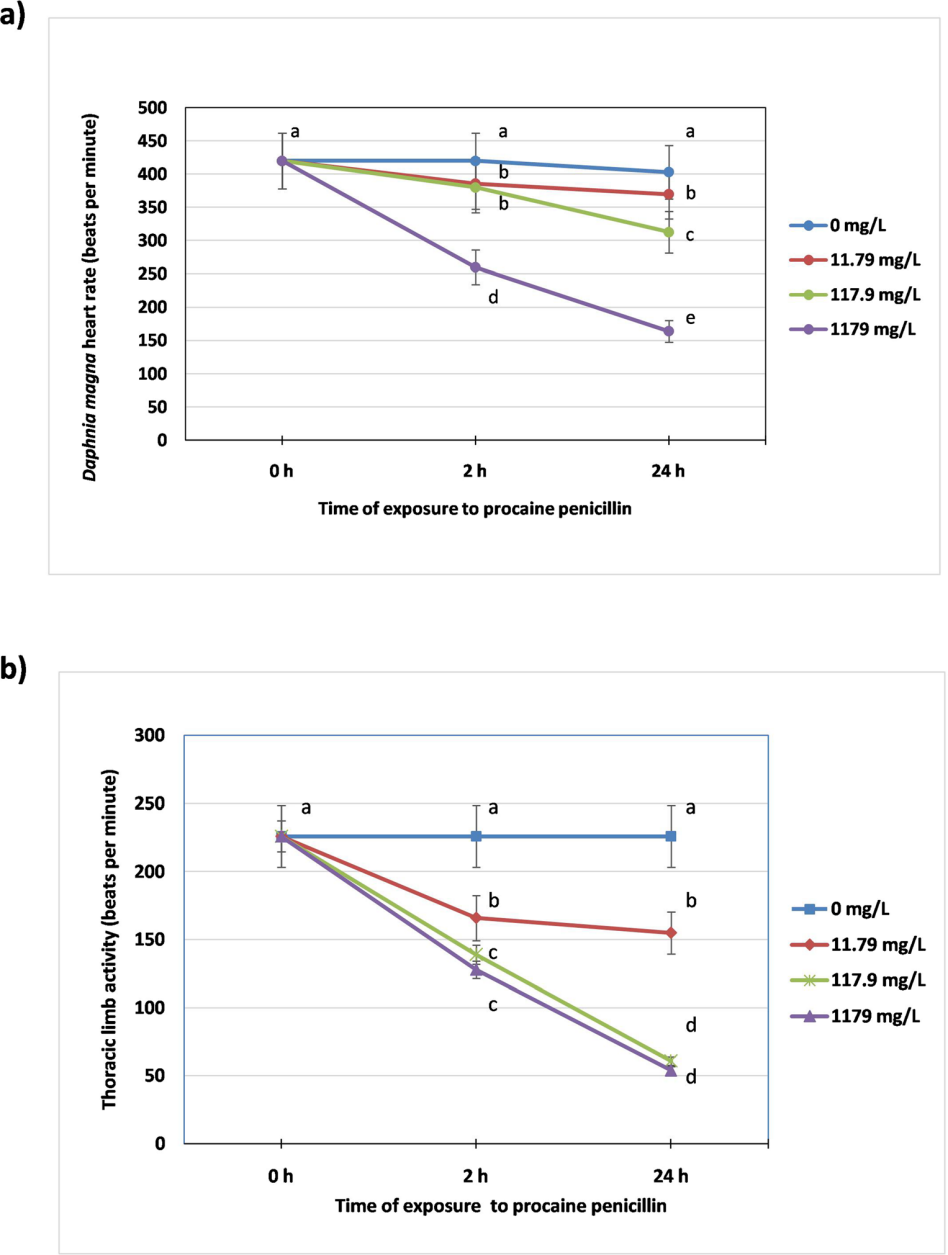
Physiological parameters

The study showed that heart rate of *Daphnia magna* exposed to PP showed a concentration-dependent inhibition after 2 and 24 h of the exposure (Fig. 7a ). The highest depression of heart contractions was found in animals after 24-h exposure to 1179 mg/L (164 ± 15 beats per minute, b.p.m.) when compared to the untreated control (420 ± 50 b.p.m.). The antibiotic at a concentration of 117.9 mg/L also considerably diminished this parameter (313 ± 20 b.p.m).

### Thoracic limb activity

Thoracic limb movement of *Daphnia magna* was affected by PP (Fig. 7b). Although a decrease of the parameter was found after 2 h of the exposure, the highest inhibition was noted in *Daphnia magna* after 24 h treatment at concentrations of 117.9 mg/L and 1179 mg/L of PP (61 ± 5 b.p.m. and 54 ± 10 b.p.m. at 117.9 mg/L and 1179 mg/L, respectively) in comparison to the control (226 ± 24 b.p.m.).

## Discussion

Some reports suggest lethal effects of various antibiotics on *Daphnia magna* (Martins et al. 2013; Ribeiro et al. 2018); however, knowledge on the influence on sensitive behavioural and physiological biomarkers of freshwater cladocerans is still very scarce. This study showed that PP affected sensitive biomarkers of swimming behaviour (swimming track density, speed, turning angle and hopping frequency), physiological parameters (oxygen consumption, heart rate and thoracic limb activity) of *Daphnia magna* in a time- and concentration-dependent manner.

### Swimming behaviour

Swimming performance of *Daphnia magna* is a sensitive and reliable biomarker with several indicators used for determination of detrimental effects of a variety of chemicals (Shimizu et al. 2002; Bownik and Stępniewska 2015; Bownik 2017; Bownik et al. 2018; Liu et al. 2018). However, the swimming behaviour is a very complex endpoint consisting of several parameters which cannot be determined quantitatively without support of digital video analysis (Bownik 2017). Moreover, one of the main disadvantages of this technique is that results may be influenced by experimental conditions such as ambient temperature, water turbidity, light or the experimental vessel size (Dodson et al. 1997; Chen et al. 2012). Behavioural responses of cladoceran to waterborne antibiotics have been poorly investigated. A study by Pan et al. (2017) revealed that *Daphnia magna* exposed to a fluorochinolone, norfloxacin increased duration of quiescence and time ratio of vertical to horizontal swimming. Our study showed that all measured swimming parameters swimming track density, hopping frequency, swimming speed and turning ability were rapidly decreased in the animals in a time- and concentration-dependent manner when compared to the control. Low water turbidity at each concentration of PP did not affect the behavioural parameters. However, it is noteworthy that the video analysis of *Daphnia* swimming behaviour was performed with the use of a 2-dimensional system which has some disadvantages when referring the results to the natural conditions. Firstly, the experimental animals kept in the observation dish with a small swimming area were forced to move between the edges which possibly resulted in the higher values of turning ability and swimming track density but the lower average swimming speed when compared to the real scenario. Secondly, since our study was performed in a 2-dimensional system with low depth of medium, the experimental animals moved horizontally with little possibility of vertical migration. Furthermore, this method did not allow us to quantify the escaping behaviour of daphnids. Despite these methodological disadvantages, the differences of swimming behaviour between the PP-treated and control animals were evident. The present study suggests that early inhibition of swimming parameters probably is a consequence of neurotoxic effects of PP. The hypothetical molecular mechanism of *Daphnia* locomotor inhibition may involve interaction of the antibiotic particularly procaine molecule with sodium channels in the motor neurons controlling muscles of the second antennae (Lin and Tsai 2005; Chen et al. 2008; Wang and Strichartz 2012). Depressive activity of procaine on this type of cells was also documented in frogs (Katz and Miledi 1975). Natural exposure to PP may result in the reduction of *Daphnia* ability to avoid predators or impair their migration for food.

### Oxygen consumption

Effects of some chemicals on the cladoceran oxygen consumption were previously determined by some authors who found the possibility of using this endpoint as a sensitive biomarker (Geiger and Buikema Jr 1981; Martins et al. 2007). Although little is known on the influence of PP or other antibiotics on the oxygen consumption in invertebrates, this parameter was studied in a mussel *Lampsilis siliquoidea* exposed to moxifloxacin; however, the results showed no alteration when compared to the untreated control (Gilroy et al. 2014). The present study showed that 2-h exposure to the lower concentrations of PP induced stimulation of oxygen consumption which may be explained by the fact that the exposure to the drug increased the oxygen demands for its enzymatic detoxification of procaine by esterases (Tobin et al. 1977; Palanivelu et al. 2005; Jewell et al. 2007; Toumi et al. 2016). On the other hand, a pronounced depression of the parameter found at the highest concentration and after 24 h at each concentration may be a result of anoxia (Hirst and Wood 1971). PP-induced depression of oxygen consumption rate was also noted in vertebrates (Vogel et al. 1957). This suggests that this parameter is a typical response to the antibiotic occurring both in the invertebrates and vertebrates and may be considered as a common biomarker

### Heart rate

*Daphnia* heart rate is a physiological biomarker of environmental stress that can be easily measured using microscopic methods (Villegas-Navarro et al. 2003; Campbell et al. 2004; Bownik 2015; Bownik and Stępniewska 2015; Bownik et al. 2019). Although the effects of antibiotics on cladoceran heart rate have not been extensively studied, Pan et al. (2017) found that norfloxacin decreased this parameter. Our study showed that PP also inhibited *Daphnia* heart rate in a time- and concentration-dependent manner. Although the mode of toxic action of norfloxacin in *Daphnia* heart has not been elucidated, PP-induced inhibition of this endpoint seems to be a result of blocking the sodium channels in the heart nerves (Frank and Sanders 1963; Dorward et al. 1983; Rosenberg et al. 1993; Yu et al. 2017). Depression of heart activity was also noted in *Daphnia magna* exposed to clove oil containing another sodium channel blocking agent, eugenol (Bownik 2015; Wang et al. 2015; Bownik 2016). Dysfunctions of heart action may lead to disturbances of haemolymph circulation resulting in reduction of oxygen supply to the organs and the increased susceptibility to various pathogens.

### Thoracic limb activity

Thoracic limbs play an important role in *Daphnia* feeding and ventilation due to generation of water currents by which food particles may be filtrated and oxygen may be supplied to the body (Pirow et al. 1999; Lari et al. 2017). Appendage activity was previously used as a sensitive biomarker (Lovern et al. 2007; Bownik and Stępniewska 2015) for determination of toxic effects induced by various compounds; however, little is known about its response to antibiotics. The present study showed that thoracic limb activity was decreased by PP in a time- and concentration-dependent manner. Depression of the parameter seems to be caused by the inhibitory action of procaine molecule on motor neurons and thus inhibitory effects on thoracic limb muscles (Katz and Miledi 1975; Ahn and Karaki 1988). Reduction of this biomarker was also found in *Daphnia magna* exposed to clove oil containing eugenol as the active ingredient (Bownik 2015; Bownik 2016). Disturbances of thoracic limb activity in cladocerans may result in reduction of ventilation leading to decreased oxygen supply to the organism or may cause impairment of feeding activity deranging the food web relations in the aquatic ecosystem.

β-lactam antibiotics are a vast group of drugs that may enter the aquatic environment from fish farms and with effluents discarded from water treatment plants (Done et al. 2015; Azanu et al. 2018; Praveena et al. 2018). Considering that the average level of these pharmaceuticals in wastewater is low (127.49 ng/L) (Kim et al. 2018) in comparison to the concentrations used in our study, constant exposure to β-lactams should not affect swimming behaviour and physiological parameters of *Daphnia magna*. However, veterinary practice of eradication of fish diseases with the intensive use of these antibiotics may elevate concentrations of antibiotics (Zhong et al. 2018) and thus pose a risk of toxic effects in cladocerans. Moreover, increased levels of β-lactams may also occur in the proximity of wastewater discharge sewers increasing the possibility of detrimental effects.

## Concluding remarks

Although our study showed no mortality of *Daphnia magna*, we found that PP affected sensitive endpoints such as swimming behaviour and physiological parameters by induction of neurotoxic changes. Since natural concentrations of PP in water reservoirs seem to be low, exposure to this antibiotic may be increased in fish farms or in the proximity of some areas where this compound is produced or intensively used. The results from the present investigation showed high sensitivity of behavioural and physiological biomarkers which suggests their recommendation for toxicological testing or monitoring of water quality.

## References

[CR1] Ahn HY, Karaki H (1988). Inhibitory effects of procaine on contraction and calcium movement in vascular and intestinal smooth muscles. Br J Pharmacol.

[CR2] American Society of Testing and Materials (1986). Standard practice for conducting static acute toxicity tests on wastewaters with Daphnia: Annual book of ASTM standards.

[CR3] Araskiewicz A, Rybakowski JK (1994). Hoigné's syndrome: a procaine-induced limbic kindling. Med Hypotheses.

[CR4] Arslan-Alaton I, Caglayan AE (2006). Toxicity and biodegradability assessment of raw and ozonated procaine penicillin G formulation effluent. Ecotoxicol Environ Saf.

[CR5] Azanu D, Styrishave B, Darko G, Weisser JJ, Abaidoo RC (2018). Occurrence and risk assessment of antibiotics in water and lettuce in Ghana. Sci Total Environ.

[CR6] Basha S, Barr C, Keane D, Nolan K, Morrissey A, Oelgemöller M, Tobin JM (2011). On the adsorption/photodegradation of amoxicillin in aqueous solutions by an integrated photocatalytic adsorbent (IPCA): experimental studies and kinetics analysis. Photochem Photobiol Sci.

[CR7] Bazakis AM, Weir AJ (2018) Procaine Penicillin. StatPearls [Internet]. Treasure Island (FL): Stat Pearls Publishing 2018-2017 Dec 21

[CR8] Bownik A (2015). Clove essential oil from Eugenia caryophyllus induces anesthesia, alters swimming performance, heart functioning and decreases survival rate during recovery of *Daphnia magna*. Turk J Fish Aquat Sci.

[CR9] Bownik A (2016). Protective effects of ectoine on physiological parameters of Daphnia magna subjected to clove oil-induced anaesthesia. Turk J Fish Aquat Sci.

[CR10] Bownik A (2017). Daphnia swimming behaviour as a biomarker in toxicity assessment: a review. Sci Total Environ.

[CR11] Bownik A, Stępniewska Z (2015). Ectoine alleviates behavioural, physiological and biochemical changes in Daphnia magna subjected to formaldehyde. Environ Sci Pollut Res Int.

[CR12] Bownik A, Sokołowska N, Ślaska B (2018). Effects of apomorphine, a dopamine agonist, on Daphnia magna: imaging of swimming track density as a novel tool in the assessment of swimming activity. Sci Total Environ.

[CR13] Bownik A, Kowalczyk M, Bańczerowski J (2019). Lambda-cyhalothrin affects swimming activity and physiological responses of *Daphnia magna*. Chemosphere.

[CR14] Campbell AK, Wann KT, Matthews SB (2004). Lactose causes heart arrhythmia in the water flea Daphnia pulex. Comp Biochem Physiol B Biochem Mol Biol.

[CR15] Chen YH, Lu KL, Hsiao RW, Lee YL, Tsai HC, Lin CH, Tsai MC (2008). Effects of penicillin on procaine-elicited bursts of potential in central neuron of snail, Achatina fulica. Comp Biochem Physiol C Toxicol Pharmacol.

[CR16] Chen L, Fu X, Zhang G, Zeng Y, Ren Z (2012). Influences of temperature, turbidity on the behavioural responses of *Daphnia magna* and Japaneese (*Oryzias latipes*) in the biomonitor. Proc Environ Sci.

[CR17] Crowe G, Theodore C, Forster GE, Goh BT (1997). Acceptability and compliance with daily injections of procaine penicillin in the outpatient treatment of syphilis-treponemal infection. Sex Transm Dis.

[CR18] Dalla Bona M, Lizzi F, Borgato A, De Liguoro M (2016). Increasing toxicity of enrofloxacin over four generations of Daphnia magna. Ecotoxicol Environ Saf.

[CR19] Dodson SI, Ryan S, Tollrian R, Lempert W (1997). Individual swimming behavior of Daphnia: effects of food, light and container size in four clones. J Plankton Res.

[CR20] Done HY, Venkatesan AK, Halden RU (2015). Does the recent growth of aquaculture create antibiotic resistance threats different from those associated with land animal production in agriculture?. AAPS J.

[CR21] Dorward PK, Flaim M, Ludbrook J (1983). Blockade of cardiac nerves by intrapericardial local anaesthetics in the conscious rabbit. Aust J Exp Biol Med Sci.

[CR22] Frank GB, Sanders HD (1963). A proposed common mechanism of action for general and local anaesthetics in the central nervous system. Br J Pharmacol Chemother.

[CR23] Geiger JG, Buikema AL (1981). Oxygen consumption and filtering rate of Daphnia pulex after exposure to water-soluble fractions of naphthalene, phenanthrene, No. 2 fuel oil, and coal-tar creosote. Bull Environ Contam Toxicol.

[CR24] Gilroy EA, Klinck JS, Campbell SD, McInnis R, Gillis PL, de Solla SR (2014). Toxicity and bioconcentration of the pharmaceuticals moxifloxacin, rosuvastatin, and drospirenone to the unionid mussel Lampsilis siliquoidea. Sci Total Environ.

[CR25] Guilhermino L, Vieira LR, Ribeiro D, Tavares AS, Cardoso V, Alves A, Almeida JM (2018). Uptake and effects of the antimicrobial florfenicol, microplastics and their mixtures on freshwater exotic invasive bivalve Corbicula fluminea. Sci Total Environ.

[CR26] Havelkova B, Beklova M, Kovacova V, Hlavkova D, Pikula J (2016). Ecotoxicity of selected antibiotics for organisms of aquatic and terrestrial ecosystems. Neuro Endocrinol Lett.

[CR27] Hirst GD, Wood DR (1971). On the neuromuscular paralysis produced by procaine. Br J Pharmacol.

[CR28] Huang DJ, Hou JH, Kuo TF, Lai HT (2014). Toxicity of the veterinary sulfonamide antibiotic sulfamonomethoxine to five aquatic organisms. Environ Toxicol Pharmacol.

[CR29] Jewell C, Ackermann C, Payne NA, Fate G, Voorman R, Williams FM (2007). Specificity of procaine and ester hydrolysis by human, minipig, and rat skin and liver. Drug Metab Dispos.

[CR30] Katz B, Miledi R (1975). The effect of procaine on the action of acetylcholine at the neuromuscular junction. J Physiol.

[CR31] Kim HY, Jeon J, Hollender J, Yu S, Kim SD (2014). Aqueous and dietary bioaccumulation of antibiotic tetracycline in D. magna and its multigenerational transfer. J Hazard Mater.

[CR32] Kim B, Ji K, Kho Y, Kim PG, Park K, Kim K, Kim Y, Kim KT, Choi K (2017). Effects of chronic exposure to cefadroxil and cefradine on Daphnia magna and Oryzias latipes. Chemosphere.

[CR33] Kim C, Ryu HD, Chung EG, Kim Y (2018). Determination of 18 veterinary antibiotics in environmental water using high-performance liquid chromatography-q-orbitrap combined with on-line solid-phase extraction. J Chromatogr B Anal Technol Biomed Life Sci.

[CR34] Laquaz M, Dagot C, Bazin C, Bastide T, Gaschet M, Ploy MC, Perrodin Y (2018). Ecotoxicity and antibiotic resistance of a mixture of hospital and urban sewage in a wastewater treatment plant. Environ Sci Pollut Res Int.

[CR35] Lari E, Steinkey D, Pyle GG (2017). A novel apparatus for evaluating contaminant effects on feeding activity and heart rate in Daphnia spp. Ecotoxicol Environ Saf.

[CR36] Lin CH, Tsai MC (2005) Effects of procaine on a central neuron of the snail, Achatina fulica Ferussac. Life Sci 76:1641–1666 10.1016/j.lfs.2004.09.020 15680172

[CR37] Liu Y, Xia C, Fan Z, Wu R, Chen X, Liu Z (2018). Implementation of fractal dimension and self-organizing map to detect toxic effects of toluene on movement tracks of *Daphnia magna*. J Toxicol.

[CR38] Lovern SB, Strickler JR, Klaper R (2007). Behavioral and physiological changes in Daphnia magna when exposed to nanoparticle suspensions (titanium dioxide, nano-C60, and C60HxC70Hx). Environ Sci Technol.

[CR39] Manakul P, Peerakietkhajorn S, Matsuura T, Kato Y, Watanabe H (2017). Effects of symbiotic bacteria on chemical sensitivity of Daphnia magna. Mar Environ Res.

[CR40] Martins JC, Saker ML, Teles LF, Vasconcelos VM (2007). Oxygen consumption by Daphnia magna Straus as a marker of chemical stress in the aquatic environment. Environ Toxicol Chem.

[CR41] Martins A, Guimarães L, Guilhermino L (2013). Chronic toxicity of the veterinary antibiotic florfenicol to Daphnia magna assessed at two temperatures. Environ Toxicol Pharmacol.

[CR42] Matozzo V, Bertin V, Battistara M, Guidolin A, Masiero L, Marisa I, Orsetti A (2016). Does the antibiotic amoxicillin affect haemocyte parameters in non-target aquatic invertebrates? The clam Ruditapes philippinarum and the mussel Mytilus galloprovincialis as model organisms. Mar Environ Res.

[CR43] Moulin G, Cavalie P, Pellanne I, Chevance A, Laval A, Millemann Y, Colin P, Chauvin C (2008). A comparison of antimicrobial usage in human and veterinary medicine in France from 1999 to 2005. J Antimicrob Chemother.

[CR44] Paine TF Jr (1978) Updating the side effects of the penicillins. Zhonghua Min Guo Wei Sheng Wu Xue Za Zhi 11: 104-109 581569

[CR45] Palanivelu V, Vijayavel K, Ezhilarasibalasubramanian S, Balasubramanian MP (2005). Impact of fertilizer (urea) on oxygen consumption and feeding energetics in the freshwater fish Oreochromis mossambicus. Environ Toxicol Pharmacol.

[CR46] Pan Y, Yan SW, Li RZ, Hu YW, Chang XX (2017) Lethal/sublethal responses of Daphnia magna to acute norfloxacin contamination and changes in phytoplankton-zooplankton interactions induced by this antibiotic. Sci Rep 12:40385 10.1038/srep40385 PMC5227989 28079143

[CR47] Park S, Choi K (2008). Hazard assessment of commonly used agricultural antibiotics on aquatic ecosystems. Ecotoxicology.

[CR48] Pellmar TC, Wilson WA (1977). Penicillin effects on iontophoretic responses in Aplysia californica. Brain Res.

[CR49] Pirow R, Wollinger F, Paul RJ (1999). The importance of the feeding current for oxygen uptake in the water flea Daphnia magna. J Exp Biol.

[CR50] Praveena SM, Shaifuddin SNM, Sukiman S, Nasir FAM, Hanafi Z, Kamarudin N, Ismail THT, Aris AZ (2018). Pharmaceuticals residues in selected tropical surface water bodies from Selangor (Malaysia): occurrence and potential risk assessments. Sci Total Environ.

[CR51] Ren X, Wang Z, Gao B, Liu P, Li J (2017). Effects of florfenicol on the antioxidant status, detoxification system and biomolecule damage in the swimming crab (Portunus trituberculatus). Ecotoxicol Environ Saf.

[CR52] Ribeiro AR, Sures B, Schmidt TC (2018). Ecotoxicity of the two veterinarian antibiotics ceftiofur and cefapirin before and after photo-transformation. Sci Total Environ.

[CR53] Richardson ML, Brown JM (1985). The fate of pharmaceutical chemicals in the aquatic environment. J Pharm Pharmacol.

[CR54] Rizzo L, Meric S, Guida M, Kassinos D, Belgiorno V (2009). Heterogenous photocatalytic degradation kinetics and detoxification of an urban wastewater treatment plant effluent contaminated with pharmaceuticals. Water Res.

[CR55] Rosenberg PH, Zou J, Heavner JE (1993). Comparison of acute central nervous system and cardiovascular toxicity of 2-chloroprocaine and prilocaine in the rat. Acta Anaesthesiol Scand.

[CR56] Shimizu N, Ogino C, Kawanishi T, Hayashi Y (2002). Fractal analysis of Daphnia motion for acute toxicity bioassay. Environ Toxicol.

[CR57] Soucek DJ (2006). Effects of freshly neutralized aluminum on oxygen consumption by freshwater invertebrates. Arch Environ Contam Toxicol.

[CR58] Soucek DJ, Dickinson A, Cropek DM (2010) Effects of millimeter wave carbon fibers on filter-feeding freshwater invertebrates. Ecotoxicol Environ Saf 73:500–506 10.1016/j.ecoenv.2009.10.015 19926133

[CR59] Taponen S, Jantunen A, Pyörälä E, Pyörälä S (2003). Efficacy of targeted 5-day combined parenteral and intramammary treatment of clinical mastitis caused by penicillin-susceptible or penicillin-resistant Staphylococcus aureus. Acta Vet Scand.

[CR60] Tobin T, Tai CY, O'Leary J, Sturma L, Arnett S (1977). Pharmacology of procaine in the horse: evidence against the existence of a “procaine - penicillin” complex. Am J Vet Res.

[CR61] Toumi H, Bejaoui M, Touaylia S, Burga Perez KF, Ferard JF (2016). Effect of carbaryl (carbamate insecticide) on acetylcholinesterase activity of two strains of Daphnia magna (Crustacea, Cladocera). J Environ Sci Health B.

[CR62] Villegas-Navarro A, Rosas-L E, Reyes JL (2003). The heart of Daphnia magna: effects of four cardioactive drugs. Comp Biochem Physiol C Toxicol Pharmacol.

[CR63] Vogel GR, Hauge SM, Andrews FN (1957). Effect of intramuscular administration of antibiotics on the oxygen consumption of normal and hyperthyroid rats. Am J Phys.

[CR64] Wang GK, Strichartz GR (2012). State-dependent inhibition of sodium channels by local anesthetics: a 40-year evolution. Biochem (Mosc) Suppl Ser A Membr Cell Biol.

[CR65] Wang ZJ, Tabakoff B, Levinson SR, Heinbockel T (2015). Inhibition of Nav1.7 channels by methyl eugenol as a mechanism underlying its antinociceptive and anesthetic actions. Acta Pharmacol Sin.

[CR66] Wollenberger L, Halling-Sørensen B, Kusk KO (2000). Acute and chronic toxicity of veterinary antibiotics to Daphnia magna. Chemosphere.

[CR67] Wright AJ (1999). The penicillins. Mayo Clin Proc.

[CR68] Yu XJ, Zhao W, Li YJ, Li FX, Liu ZJ, Xu HL, Lai LY, Xu R, Xu SY (2017). Neurotoxicity comparison of two types of local anaesthetics: amide-bupivacaine versus ester-procaine. Sci Rep.

[CR69] Zhong Y, Chen ZF, Dai X, Liu SS, Zheng G, Zhu X, Liu S, Yin Y, Liu G, Cai Z (2018). Investigation of the interaction between the fate of antibiotics in aquafarms and their level in the environment. J Environ Manag.

